# Breast milk mesenchymal stem cells abate cisplatin-induced cardiotoxicity in adult male albino rats via modulating the AMPK pathway

**DOI:** 10.1038/s41598-022-22095-2

**Published:** 2022-10-20

**Authors:** Mahitab M. Nageeb, Sara F. Saadawy, Seba Hassan Attia

**Affiliations:** 1 Clinical Pharmacology Department, Faculty of Medicine, Zagazig University, Zagazig, Egypt; 2 Medical Biochemistry Department, Faculty of Medicine, Zagazig University, Zagazig, Egypt

**Keywords:** Biochemistry, Stem cells, Cardiology, Diseases

## Abstract

Myocardial injury influenced by cisplatin (Cis) is a compelling reason to hunt out a treatment modality to overcome such a threat in cisplatin-treated patients. Breast Milk mesenchymal stem cells (Br-MSCs) are a non-invasive, highly reproducible source of stem cells. Herein, we investigate Br-MSCs' role in cardiotoxicity induced by cisplatin. Rats were divided into; control, Cis-treated (received 12 mg/kg single intraperitoneal injection), BrMSCs-treated (received single intraperitoneal injection of 0.5 ml sterilized phosphate-buffered saline containing 2 × 10^7^ cells of Br-MSCs); metformin-treated (received 250 mg/kg/day orally) and BrMSCs + metformin + Cis treated groups. At the experiment end, serum creatine kinase (CK-MB) and cardiac troponin T (cTnT) activates were estimated, cardiac malondialdehyde (MDA), superoxide dismutase (SOD), interleukin-1ß (IL-1ß), tumor necrosis factor-α (TNF-α) levels were measured, cardiac expression of Bax and Bcl-2 and AMP-activated protein kinase (AMPK), as well as heart histopathology, were evaluated. Study results showed that Cis explored acute cardiotoxicity evidenced by deteriorated cardiac indices, induction of oxidative stress, and inflammation with myocardium histological alterations. Treatment with Br-MSCs restored heart function and structure deteriorated by Cis injection. The antioxidant/anti-inflammatory/anti-apoptotic results of Br-MSCs were supported by AMPK activation denoting their protective role against cisplatin-induced cardiac injury. These results were superior when metformin was added to the treatment protocol.

## Introduction

Cisplatin (Cis), a clinically potent broad-spectrum platinum-based anticancer drug, has a robust antineoplastic potential against numerous malignancies including ovarian, testicular, breast, lung, head, and neck tumors by targeting the DNA replication of tumor cells^1–3^. Despite the comprehensive tumor progression restriction, the clinical use of cisplatin is limited as it seriously affects the internal organs which threatens patients’ life^4^. Cardiotoxicity, either acute or cumulative, is one of the clinical drawbacks of cisplatin, which is characterized by many clinical events manifested as a midrange ejection fraction, and dysrhythmias^5^ along with myocarditis, cardiomyopathy, and alterations in the Electrocardiogram (ECG)^2^. Therefore, implementing a balance between cisplatin efficacy and cardiotoxicity is crucial to attaining the optimal anticancer drug action in parallel with abating cardiotoxicity which is a non-avoidable clinical issue^6^. Understanding the underlying molecular mechanisms of cisplatin-induced cardiotoxicity is mandatory for dwindling organ damage. As reported in the literature, the production of reactive oxygen species (ROS) after exposure to cisplatin triggers the oxidation of vital biological cellular molecules including proteins, plasma membrane, and nucleic acids resulting in cardiac cell DNA damage and apoptosis^7^. Moreover, El-Hawwary and Omar (2019), highlighted the role of endoplasmic reticulum (ER) stress initiated by oxidative stress, which was evoked by cisplatin intake, in the implication of cardiac cell pathogenesis^8^. It is worth noting that, myocardial injury of cisplatin could also be due to influencing the phosphorylation condition of proteins involved in the phosphatidylinositol-3-hydroxy kinase/protein kinase B (PI3K/Akt) pathway^6^.

Transplantation of stem cells is one of the outstanding modalities for treating many disorders because of their potent anti-inflammatory, anti-apoptotic and immunomodulatory properties^9–11^. They are two main types: embryonic and adult stem cells^12^. The adult type could be hematopoietic, endothelial, olfactory, neural, or mesenchymal stem cells (MSCs)^13,14^. MSCs proved their vast effectiveness in ameliorating many disorders, such as; spinal cord injury^15^, testicular dysfunction^14^, necrotizing enterocolitis^16^, cardiomyopathy^17^, and myocardial infarction^18^. Breast milk is a modern source of various types of stem cells including breast milk mesenchymal stem cells (Br-MSCs), mammary stem cells, and small embryonic-like stem cells^19^. Br-MSCs are highly reproducible having the ability to differentiate rapidly into many cell types either in vivo or in vitro producing abounding amounts of trophic substances as vascular endothelial growth factors (VEGF)^14^ and considered a non-invasive source of stem cells in comparison to other types^20^. Although the effects of Br-MSCs were scarcely investigated on cardiovascular diseases, their beneficial roles on different disorders have been elucidated in many kinds of literature. Khamis and colleagues found that Br-MSCs restored the ß-cell function in type-1 diabetic rats via remodeling endoplasmic reticulum stress and inflammatory and apoptotic signaling pathways^21^. Moreover, the development of diabetic induced-testicular dysfunction and male infertility were found to be attenuated after Br-MSCs transplantation^14,22^. In the same context, Br-MSCs have demonstrated their immunomodulating protective role in necrotizing enterocolitis^23^.

Metformin, well tolerated universally used anti-diabetic treatment for type-2 diabetes, showed its effectiveness in ameliorating the risk of cardiovascular diseases^24^ and reducing heart failure patients’ mortality^25^**.** The mechanism by which metformin increases the cardiomyocyte survival rate was known to be through the activation of 5′-adenosine monophosphate-activated protein kinase (AMPK) which subsequently reduces ROS generation^26^.

Based on the past view about the cardiotoxic hazardous sequences that develop after cisplatin injection in cancer patients treated with such anti-tumor drug, the seek for different pharmacological manners to overcome cisplatin induced carditoxicity became crucial. So, this study was constructed to investigate the potential value of Br-MSCs alone and in combination with metformin in attenuating cisplatin-induced cardiotoxicity as well as to highlight the involved underlying molecular mechanisms. It is worth noting that, to date research on this point is still relatively shallow.

## Materials and methods

### Experimental animals

Fifty adult male albino rats were used in the experiment. Rats were purchased from the Faculty of Veterinary Medicine, Zagazig University, Egypt. Animals were left under precise pathogen-free conditions for one week as an acclimatization period; standard humidity, 12 h light/dark cycles, and a temperature of 22 ± 2 °C. Standard pelleted food and water were given ad libitum to rats. The study was conducted in the animal house of the Faculty of Medicine, Zagazig University.

### Ethical statement

The study protocol was approved by the Institutional Animal Care and Use Committee at the Faculty of Medicine, Zagazig University, Egypt (ZU-IACUC/3/F/178/2021), and experimentation was also followed by the National Institutes of Health Guide for care and use of laboratory animals (NIH Publications “No.8023, revised 1996”). All experimental procedures complied with recommendations in ARRIVE guidelines.

### Study design

The experiment was conducted on fifty adult Wistar albino male rats that were assigned randomly into five groups (10 rats each) as follows:

*Group I (Control group);* rats were injected with an equal volume of saline once followed by daily administration of saline orally for 14 days.

*Group II (Cisplatin group);* received a single intraperitoneal (i.p) injection dose (12 mg/kg)^27^ of cisplatin and followed by daily administration of saline orally for 14 days.

*Group III (BrMSCs* + *Cis treated group);* received a single i.p injection dose (12 mg/kg) of cisplatin followed by an intraperitoneal injection of 0.5 ml sterilized phosphate-buffered saline (PBS) containing 2 × 10^7^ cells of Br-MSCs^21^ followed by daily administration of saline orally for 14 days.

*Group IV (metformin* + *Cis treated group);* received a single i.p injection dose (12 mg/kg) of cisplatin then treated with 250 mg/kg/day metformin, orally by gavage for 14 days^28^.

*Group V(BrMSCs* + *metformin* + *Cis treated group);* received a single i.p injection dose (12 mg/kg) of cisplatin then treated by intraperitoneal injection of 0.5 ml PBS containing 2 × 10^7^ cells of Br-MSCs + 250 mg/kg/day metformin, orally by gavage for 14 days.

### Collection, preparation, culturing and identification of Br-MSCs

#### Collection of Br-MSCs (ethical approval)

The milk samples were collected as aseptically as possible from the breastfeeding unit in the Department of Pediatrics, Zagazig University Hospitals in accordance with relevant guidelines and regulations. All experimental procedures were approved and formed in accordance with the guidelines of the institutional review board (IRB#: 9931-7-11-2021), Faculty of Medicine, Zagazig University, Egypt. The milk sample collection was performed after taking informed written consent from nursing mothers two to five days post-partum according to Patki and colleagues’ method^56^.

#### Preparation, culturing of Br-MSCs

The preparation and culturing procedures were performed according to the protocol described by Patki et al.^29^. Briefly, breast milk was diluted 1:2 with high glucose DMEM (4.5 g/L glucose with L-glutamine, Lonza Bioproducts Walkersville, MD 21793–0127 USA) containing a mixture of penicillin–streptomycin- Amphotericin B as 10 IU/10 IU/25 mg (Lonza Bioproducts Walkersville, MD 21793–0127 USA) and centrifuged at 285 *g* for 10 min. The cell pellet was washed twice with sterile phosphate-buffered saline (PBS) (Lonza Bioproducts Walkersville, MD 21793–0127 USA). The final cell pellet was seeded in 25 cm2 tissue culture flasks containing DMEM medium, 10% fetal bovine serum (FBS), and penicillin–streptomycin-amphotericin B (Lonza Bioproducts Walkersville, MD 21793–0127 USA).

The flasks were incubated at 37 °C under 5% CO2 and 95% relative humidity in a CO2 incubator (Heraeus, Hanau, Germany). The medium was changed every 48 h for 14 days as primary passage. At confluency of 80%, cells were trypsinized with 0.25% trypsin containing 0.02% EDTA (Lonza Bioproducts) for 5 min at 37 °C and centrifuged at (2400 rpm for 20 min). Cells were counted with a hemocytometer and their viability was conducted with a trypan blue stain. The washed cell pellet was re-suspended in DMEM before transplant.

#### MSCs identification

**Figure 1:**
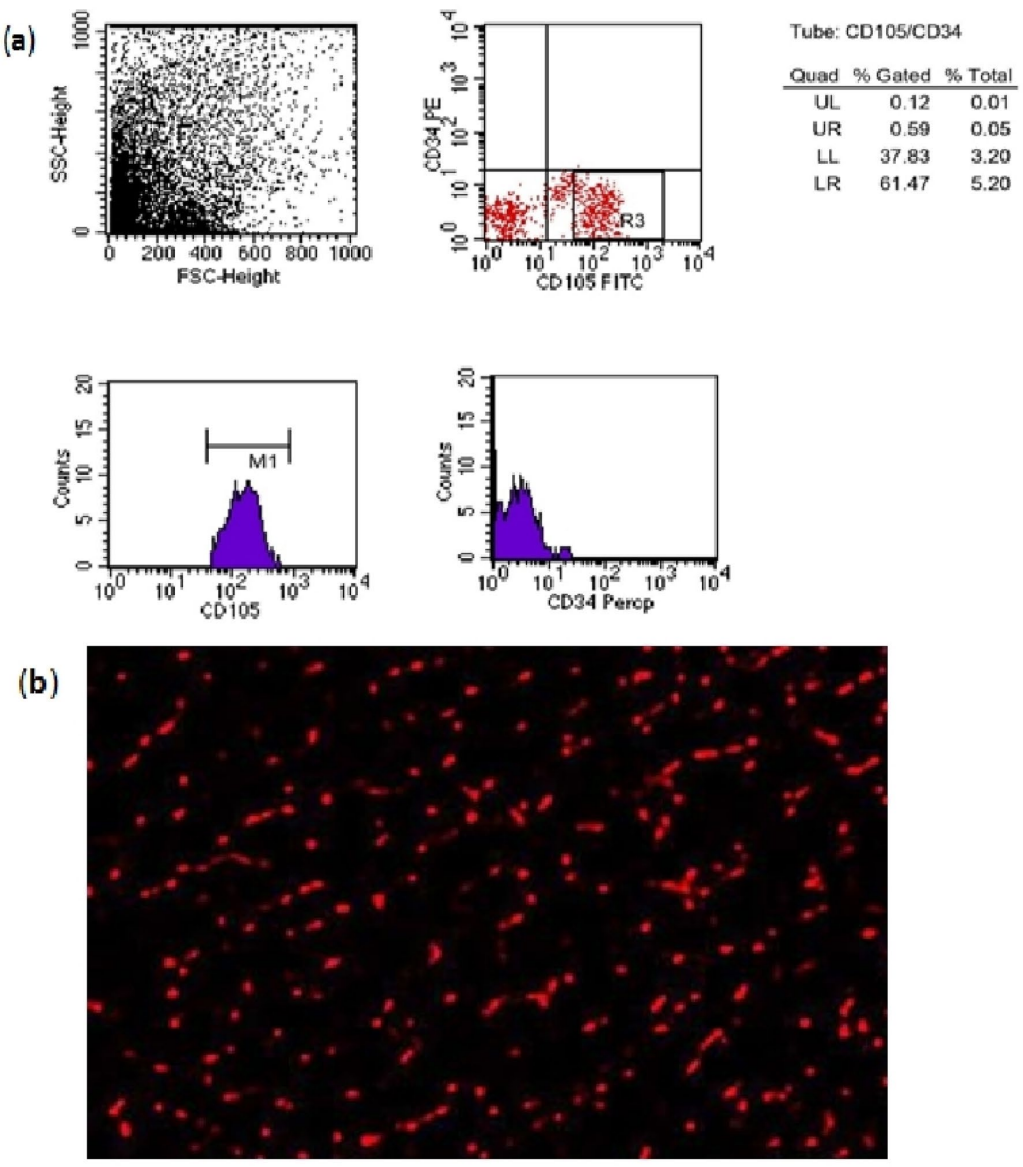
(**a**) Flow cytometry chart showing positive MSCs for CD105 and negative MSCs for CD34. (**b**) Representative fluorescence micrograph of cardiac tissue from cisplatin and breast milk MSc (Br-MSCs) treated group following MSc dosing displaying the homing of MSc by PKH26 dye.

Br-MSCs were identified according to Patki et al.^29^ methods based on their adhesiveness to the bottom of the culture flask and their fusiform, star, or spindle shape under an inverted microscope. Also, MSCs were immunophenotyped with flow cytometry. The isolated cells were positive for MSCs surface marker CD105, whereas were negative for hematopoietic marker CD34 (Fig. 1a). Before injection, The PKH26 Fluorescent Cell Linker Kit (Sigma–Aldrich) was used to label MSCs for identification of MSCs homing in cardiac tissues (Fig. 1b).

### Drugs and the rational of dose selection

Cisplatin and metformin hydrochloride were purchased from Sigma/Aldrich Chemical Co. (Merck Life Science, Gillingham, UK). Unless otherwise noted, in this experiment all other used chemicals, of analytical grade, were obtained from Sigma/Aldrich. The dose of cisplatin was selected based on the lethal dose 50 (LD50) of the intraperitoneal drug injection in rats which is 12 mg/kg^27^. Metformin dose was chosen based on the humans and rats’ pharmacokinetic formula^28,30^.

### Recording of the electrocardiogram (ECG)

At the end of the study period, rats’ body weights were determined then pentobarbital 60 mg/kg (Sigma, St. Louis, USA) was intraperitoneally injected^31^ to anesthetize the rats before carotid artery cannulation to assess ECG changes and heart rate via using the Power Lab (4/35) Data Acquisition System (Australia). Briefly, according to the method of Parasuraman & Raveendran^32^ insertion of one end of the cannula into the carotid artery of the anesthetized animals was done however the other end has been connected to a three-way stopcock. The three-way stopcock acts as a connection bridge between the carotid cannula and the pressure transducer. The three-way stopcock was also connected to a syringe filled with heparinized saline to maintain helped the positive pressure at the baseline level, all measurements were analyzed by the Lab chart seven software. After recording heart rates, QRS Interval, QTc duration, ST segment Height, and T wave amplitude, in clean dry tubes, blood samples were withdrawn from the inserted carotid cannula after that rats were sacrificed.

### Sampling and tissue dissection

Shortly, after the animal’s sacrification, the separation of sera by centrifugation at 3000 rpm for 10 min from blood samples was done. Hearts were excised, washed with saline, and then weighted to calculate the heart weight/body weight ratio (HW/BW% ratio), followed by each organ division into two equal parts; one was homogenized for bioassay of biochemical parameters; however, the other was retained in 10% formalin for the histopathological examination.

### Serum cardiac biochemical parameters analysis

Blood samples collected were centrifuged at 4 °C and sera were separated. Commercially available kits were used to measure the serum troponin T content as well as creatine kinase-MB (CK-MB) activities (Colorimetric Cat no: ab155901).

### Assay of cardiac lipid peroxidation and antioxidant enzyme

The cardiac content of malondialdehyde (MDA) was determined using the thiobarbituric acid method Liu et al.^33^. The biodiagnostic kit (catalog no. MD 2529) was used according to the manufacturer’s instructions. However, the activity of the antioxidant enzyme superoxide dismutase (SOD) was measured according to Marklund and Marklund^34^ method. The difference in the absorbance of the color at 430 nm at 0 min and after 10 min was recorded to calculate the enzyme activity using a biodiagnostic kit (catalog no. SD 25 21).

### Interleukin-1 beta and tumor necrosis factor-alpha measurements in tissue

Interleukin-1 beta and tumor necrosis factor-alpha concentrations in the tissue homogenate were measured using the following rat-specific sandwich enzyme-linked immunosorbent assay kits: Rat Interleukin 1β ELISA Kit (Cat no: ab100768) and Rat Tumor Necrosis Factor α ELISA kits (Cat no: ab100785). Analyses were performed according to the manufacturers’ instructions.

### Gene expression studies by real-time PCR (qPCR)

Total RNA was extracted from 30 mg of rat cardiac tissue with Trizol reagent (Thermo Fisher Scientific; Waltham, MA, United States). The NanoDrop spectrophotometer was used to determine the concentration and purity of the extracted RNA by estimating the OD at 260 and 280 nm and accepting A260/A280 at a ratio of 1.8–2.1 followed by a two-step real-time PCR to evaluate gene expression as reported previously^35^. Briefly, cDNA synthesis was used by a Hi Sen Script RH (−) cDNA Synthesis Kit (iNtRON Biotechnology Co., South Korea), and The real-time RT-PCR was performed in an Mx3005P Real-Time PCR System (Agilent Stratagene, USA) using 10 µl qPCR 2X PreMIX (SYBR Green with low ROX) (Cat. # P725 or P750) (Enzynomics, Korea) following the manufacturer’s instructions with specific primers for BAX, Bcl-2 and AMPK in Supplenetary Table (S1). In concentration 1 µl form Forward primer (10 pmol/µl) and 1 µl form reverse primer (10 pmol/µl) and 5 µl cDNA template then H2O PCR grade Up to total volume 20 µl. With PCR cycling conditions consisted of initial denaturation at 95 °C for 12 min, followed by 40 cycles of denaturation at 95 °C for 30-s annealing at 60 °C for 60 s, and extension at 72 °C for 60 s. The relative expression level of the target genes was normalized to that of the housekeeping Glyceraldehyde-3-phosphate dehydrogenase (GAPDH), and the relative fold changes in gene expression were calculated based on the 2 − ΔΔCT comparative method by Livak and Schmittgen^36^**.**

### Heart histological assessment

The cardiac specimens fixed in 10% formalin were embedded in paraffin wax and then processed for staining. Sections were cut at a thickness of 4 μm and stained using Hematoxylin and Eosin (H&E). Under light microscopy, the slides were examined to evaluate the severity of cardiac tissue injury according to a semi-quantitative score depending on the degree of histopathological alteration based on the degree of cardiac muscles architecture disruption, loss of muscular striations, myocyte degeneration, fibrosis and inflammatory cellular infiltrate^37^.

### Data analysis

Data were checked, entered, and analyzed by using SPSS software (Version 22.0) (SPSS Inc, Chicago, USA). Statistical difference between the studied groups was evaluated by one-way analysis of variances (one-way ANOVA) and followed by post hoc least significant difference (LSD) for multiple comparisons. The results were expressed as mean ± standard deviation (SD) and when the p-value is < 0.05 differences were considered statistically significant.

## Results

### Effect of Br-MSC, metformin, and their combination on rats’ body weight (BW), heart weight (HW), and heart weight/body weight ratio (HW/BW)

Cisplatin treatment significantly (P < 0.05) decreased rats’ BW as compared to rats’ BW in the normal control group (230.6 ± 7.96 vs. 256.8 ± 10.04 respectively). However, treatment with Br-MSC and metformin either alone or in combination significantly (P < 0.05) increased the BW of rats to be normalized in the combination group. The heart weight and heart weight to body weight ratio (HW/BW )in cisplatin-treated animals were significantly (P < 0.05) decreased in comparison to normal control animals. On the other hand, rats’ HW and the ratio of heart weight to body weight were significantly (P < 0.05) increased after treatment with Br-MSC and/or metformin. Interestingly, it was found that significantly (P < 0.05) higher values of rats’ HW and HW/BW were seen in the group of rats that received Br-MSC + metformin when compared to the group of rats treated with Br-MSC alone, denoting that adding metformin to the treatment protocol with Br-MSC had superior results than using Br-MSC alone (Table 1).

**Table 1:** Effect of Br-MSCs, metformin and their combination on body weight (BW), heart weight (HW), and heart weight/body weight ratio (HW/BW) in adult male albino rats with cisplatin-induced cardiotoxicity.

Groups	Body weight (BW) (gm)		Heart weight (HW)(gm)	Ratio (HW/BWx10^–3^)
	Initial	Final		
Control	222.7 ± 6.45	256.8 ± 10.04	0.65 ± 0.04	2.53
Cisplatin treated	221.8 ± 8.19	230.6 ± 7.96*	0.51 ± 0.02*	2.21*
Br- MSCs + Cis	219.6 ± 6.93	246.3 ± 8.98*^+^	0.60 ± 0.03^+^	2.43^+^
Metformin + Cis	224.1 ± 9.43	242.7 ± 7.93*^+^	0.62 ± 0.05^+^	2.55^+^
Br-MSCs + Metformin + Cis	220.8 ± 9.35	252.5 ± 9.14^+^	0.64 ± 0.04^+$^	2.54^+$^

*Cis* cisplatin. *Br-MSCs* Breast Milk mesenchymal stem cells.

*P < 0.05 when compared with control, ^+^ P < 0.05 when compared with cisplatin.

^$^P < 0.05 when compared with Br-MSCs + cisplatin. Statistical analysis was done by using one way ANOVA test. Values were expressed as (mean ± SD).

### Effect of Br-MSC, metformin, and their combination on heart rate and electrocardiogram (ECG) pattern

**Figure 2:**
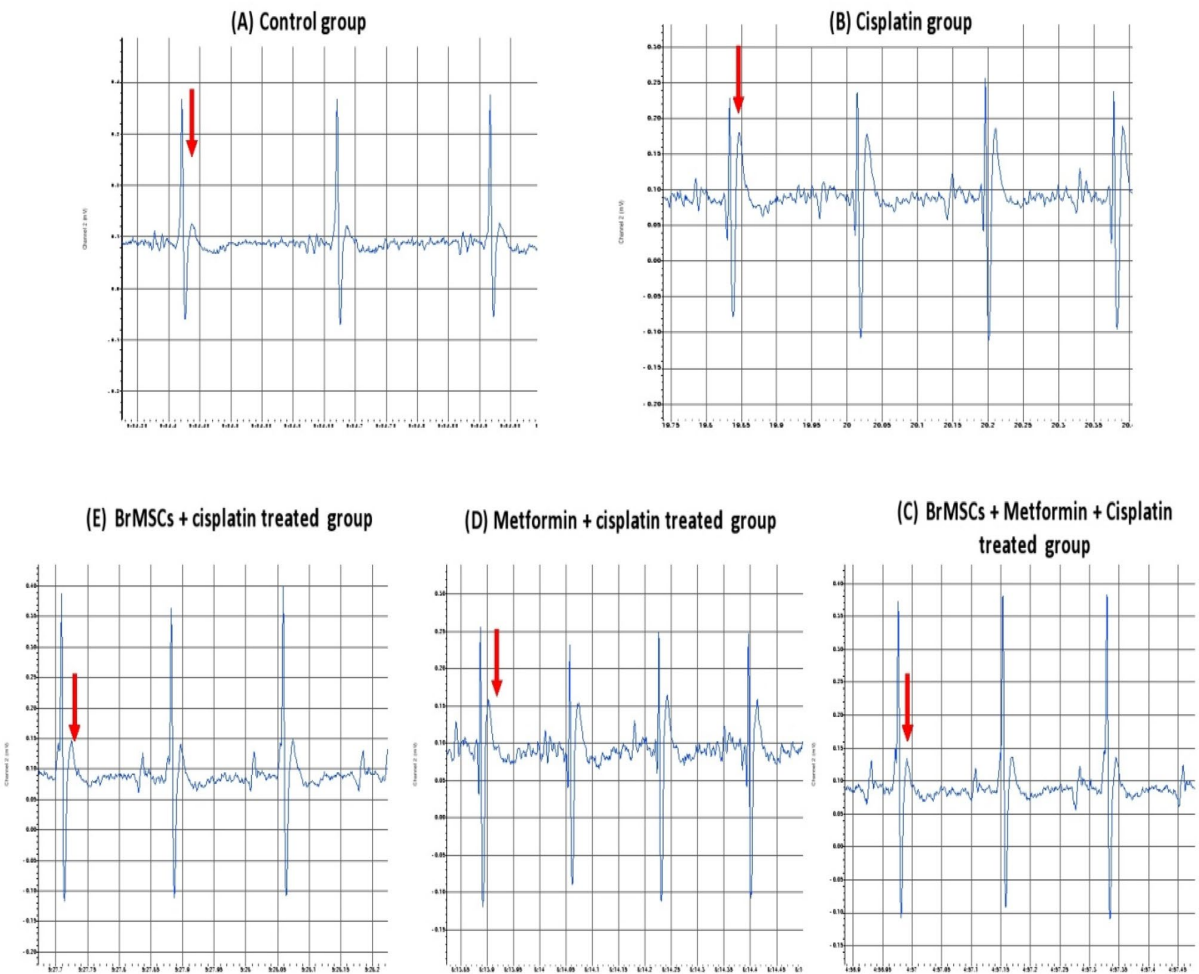
Effect of Br-MSCs, metformin, and their combination on ECG pattern.

**Figure 3:**
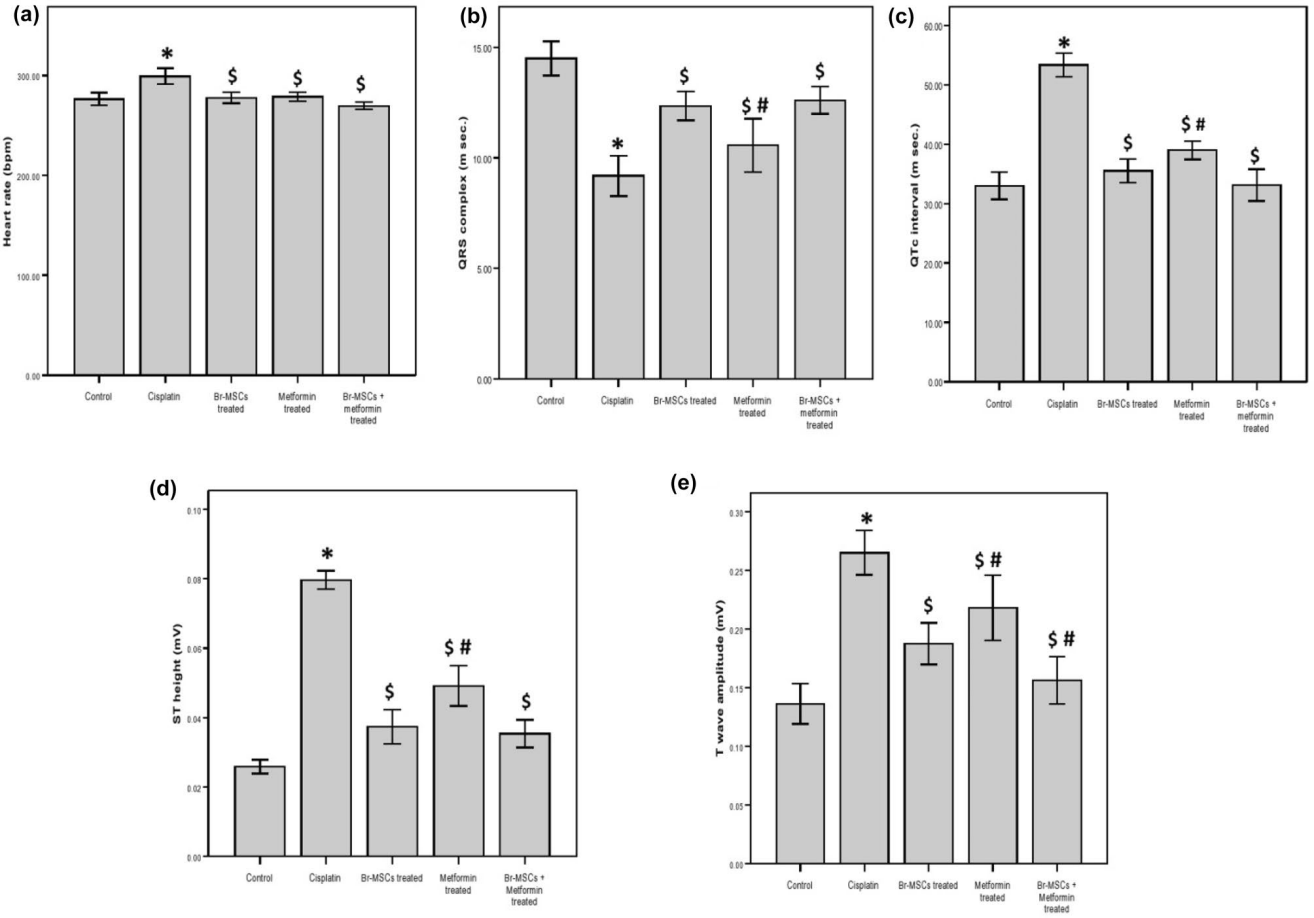
Effect of Br-MSCs, metformin, and their combination on (**a**) heart rate (**b**) QRS complex (msec.) (**c**) QTc interval duration (msec.) (**d**) ST segment height (**e**) T wave amplitude. Statistical analysis was done by using one way ANOVA test. Values were expressed as (mean ± SD). *P < 0.05 when compared with control, ^**$**^P < 0.05 when compared with cisplatin, ^#^P < 0.05 when compared with Br-MSCs + cisplatin.

As seen in (Figs. 2,3) cisplatin injection was accompanied by a significant (P < 0.05) increase in rats’ heart rate in comparison to normal rats’ heart rate. Moreover, cisplatin intoxication led to marked ECG changes when compared to control rats’ ECG pattern as there is a significant (P < 0.05) reduction in the QRS complex interval with elongation of QTc interval duration as well as increased ST height and T wave amplitude. However, rats treated with Br-MSCs alone or metformin alone or their combination significantly (P < 0.05) restored the heart rate to normal with marked improvements in the ECG pattern evidenced by significant elevation of the QRS complex interval duration with a marked reduction in QTc interval duration, ST segment height and T wave amplitude. And while comparing the group of rats that received the combined therapy and the group treated with Br-MSCs alone, there were significant (P < 0.05) improvements in the ECG pattern, specifically T wave amplitude reduction, revealing the good impact of co-administration of metformin and Br-MSC.

### Effect of Br-MSCs, metformin, and their combination on serum cardiac enzymes

As shown in Table 2 data revealed that serum CK-MB activity and cTnT plasma levels after administration of a single cisplatin dose (12 mg/kg) were significantly (P < 0.05) elevated relative to the normal control group. The average cTnT concentration was tripled, while the average CK-MB showed a sharp increase of more than 4 folds in cisplatin-treated animals, which indicates that marked cardiac injury has occurred after cisplatin administration. In contrast, rats treated with Br-MSC, metformin, and their combination induced a significant reduction (P < 0.05) in serum cardiac enzyme activities and cTnT concentration. It is noteworthy that the Br-MSC + metformin group has normalized the cisplatin-induced increase in serum CK-MB activity and cTnT plasma levels revealing significant (P < 0.05) low levels in comparison to treatment with Br-MSC alone. This suggests that the former protective treatment with Br-MSC significantly antagonized cisplatins’ toxic effect especially when metformin was added to the treatment protocol.

**Table 2:** Effect of Br-MSCs, metformin and their combination on the cardiac enzymes (cTnT and CK-MB), lipid peroxidation markers (MDA and SOD), and the pro-inflammatory cytokines (TNF-α and IL-1β) in adult male albino rats with cisplatin-induced cardiotoxicity.

Groups	Control	Cisplatin treated	Br- MSCs + cisplatin	Metformin + cisplatin	Br- MSCs + metformin + cisplatin
cTnT (ng/ml)	0.24 ± 0.04	0.61 ± 0.16*	0.32 ± 0.02*^+^	0.35 ± 0.02*^+^	0.25 ± 0.028^+$^
CK MB(Iu/L)	545.2 ± 21	2635 ± 19*	767.5 ± 56.3*^+^	737 ± 21*^+^	597.9 ± 49*^+$^
MDA (nmol/g)	27.9 ± 1.7	68 ± 8.9*	54.6 ± 2.6*^+^	53.4 ± 2.6*^+^	52.5 ± 2.3*^+^
SOD (u/mg)	0.89 ± 0.05	0.398 ± 0.05*	0.78 ± 0.08*^+^	0.7 ± 0.075*^+^	0.82 ± 0.06*^+$^
IL-1β (pg/ml)	1.9 ± 0.5	6.78 ± 1.5*	3.24 ± 0.8*^+^	3.5 ± 0.8*^+^	2.4 ± 0.6^+$^
	1.1–2.54	4.56–8.7	1.98–4.3	2.1–4.5	1.65–3.1
TNF-α (pg/ml)	2.97 ± 0.4	8.3 ± 1.2*	3.5 ± 0.6*^+^	4.38 ± 1.1*^+^	3.06 ± 0.6^+$^
	2.2–3.56	5.78–9.5	2.45–4.5	2.23–5.87	1.9–3.66

Statistical analysis was done by using one way ANOVA test.

Values were expressed as (mean ± SD).

*Br-MSCs* Breast Milk mesenchymal stem cells, *cTnT* cardiac troponin, *CK-MB* serum creatine kinase, *MDA* Malondialdehyde, *SOD* Superoxide dismutase enzyme, *TNF-α* Tumor necrosis factor-alpha**,** *IL-1β* Interleukin-1-β.

*P < 0.05 when compared with control.

^+^P < 0.05 when compared with cisplatin.

^$^P < 0.05 when compared with Br-MSCs + cisplatin.

### Effect of Br-MSCs, metformin, and their combination on lipid peroxidation

Oxidative stress is one of the major contributors to cisplatin toxicity. The present study showed that cisplatin injection was associated with significantly (P < 0.05) higher levels of malondialdehyde (MDA) compared to the control group. However, rats treated with Br-MSC alone or metformin alone or their combination revealed significant (P < 0.05) lower levels of MDA in comparison to the cisplatin group. Comparing Br-MSC + metformin + cis treated group with Br-MSC + cis treated group revealed no significant difference (Table 2).

Regarding superoxide dismutase (SOD) activity, rats acutely exposed to cisplatin showed a significant (P < 0.05) decrease in SOD activity in comparison to the control group. On the other hand, treatment with Br-MSCs and metformin either alone or in combination significantly (P < 0.05) reversed the decrease in SOD levels in the heart tissues. The SOD levels were nearly normalized in the combination group that received both Br-MSCs and metformin which showed significantly (P < 0.05) higher values compared to rats treated with Br-MSCs alone (Table 2).

### Effect of Br-MSCs, metformin, and their combination on inflammatory markers

To evaluate the possible inflammatory process secondary to cisplatin administration we estimated the levels of the pro-inflammatory cytokines; tumor necrosis factor-alpha (TNF-α) and interleukin-1-β (IL-1β). The results showed that cisplatin injection was associated with significantly (P < 0.0001) higher levels of TNF-α and IL-1β compared to the control group. On contrary, TNF-α and IL-1β levels were significantly (P < 0.0001) lower in the groups treated with Br-MSC, and metformin either alone or in combination in comparison to the cisplatin group. Interestingly, TNF-α and IL-1β levels showed no statistically significant difference between the group of rats administered both Br-MSC + metformin and the control rats. Moreover, TNF-α and IL-1β showed significantly (P < 0.05) lower values in Br-MSC + Met treated animals compared with Br-MSC treated animals, denoting the beneficial results after adding metformin to Br-MSCs (Table 2).

### Effect of Br-MSCs, metformin and their combination on cardiac tissue Bax and Bcl-2 quantitative real time-PCR gene expression

The Real-Time quantitative PCR results revealed that the anti-apoptotic gene, **Bcl-2** expression was 0.15 ± 0.07 in the cisplatin-treated group which was statistically significantly lower (P < 0.05) compared not only to the control group but also to Br-MSCs treated, metformin-treated and Br-MSCs + metformin treated groups (0.77 ± 0.24, 0.77 ± 0.2 and 0.81 ± 0.18) respectively.

Meanwhile, the expression of the pro-apoptotic gene, **Bax,** was 3.5 ± 0.7 in the cisplatin-treated group which was significantly (P < 0.05) higher in comparison to Br-MSCs treated, metformin-treated, and Br-MSCs + metformin-treated groups (2.11 ± 0.24, 2.16 ± 0.3, and 2.0 ± 0.3) respectively as well as to control group (Table 3).

**Table 3:** Effect of Br-MSCs, metformin, and their combination on the cardiac pro-apoptotic BAX gene; anti-apoptotic Bcl-2 and AMPK in adult male albino rats with cisplatin-induced cardiotoxicity.

Groups	Control	Cisplatin treated	Br- MSCs + cisplatin	Metformin + cisplatin	Br- MSCs + metformin + cisplatin
Bax	1.0	3.5 ± 0.7*	2.11 ± 0.24*^+^	2.16 ± 0.3*^+^	2.0 ± 0.3*^+^
Bcl-2	1.0	0.15 ± 0.07*	0.77 ± 0.24*^+^	0.77 ± 0.2*^+^	0.81 ± 0.18*^+^
Bax/Bcl-2 Ratio	1.0	5 ± 10*	2.74 ± 1*^+^	2.8 ± 1.5*^+^	2.5 ± 1.67*^+^
AMPK	1.0	0.47 ± 0.12*	0.88 ± 0.05*^+^	0.87 ± 0.055*^+^	0.91 ± 0.06*^+^

Statistical analysis was done by using one way ANOVA test.

Values were expressed as (mean ± SD).

*Br-MSC* Breast Milk mesenchymal stem cells.

*P < 0.05 when compared with control.

^+^P < 0.05 when compared with cisplatin.

The overexpression of the cardiac pro-apoptotic Bax gene and low expression of cardiac anti-apoptotic Bcl-2 gene with cisplatin was reflected in the Bax/Bcl-2 ratio that was significantly (P < 0.05) elevated in cisplatin-treated rats to reach 5 compared to the normal control group. However, in comparison to the cisplatin-treated group Bax/Bcl-2 ratio has decreased in Br-MSCs treated, metformin-treated and Br-MSCs + metformin treated groups (2.74 ± 1, 2.8 ± 1.5, 2.5 ± 1.67) respectively.

### Effect of Br-MSCs, metformin and their combination on AMPK an autophagic marker

Cisplatin intoxicated rats showed that AMPK gene expression by the Real-Time quantitative PCR was 0.47 ± 0.12 which was statistically significantly lower (P < 0.05) in comparison to the control group. On the other hand, treatment with Br-MSCs and metformin either alone or in combination revealed significantly (P < 0.05) higher AMPK gene expression rates (0.88 ± 0.05, 0.87 ± 0.055 and 0.91 ± 0.06) respectively (Table 3).

### Histopathological results

**Figure 4:**
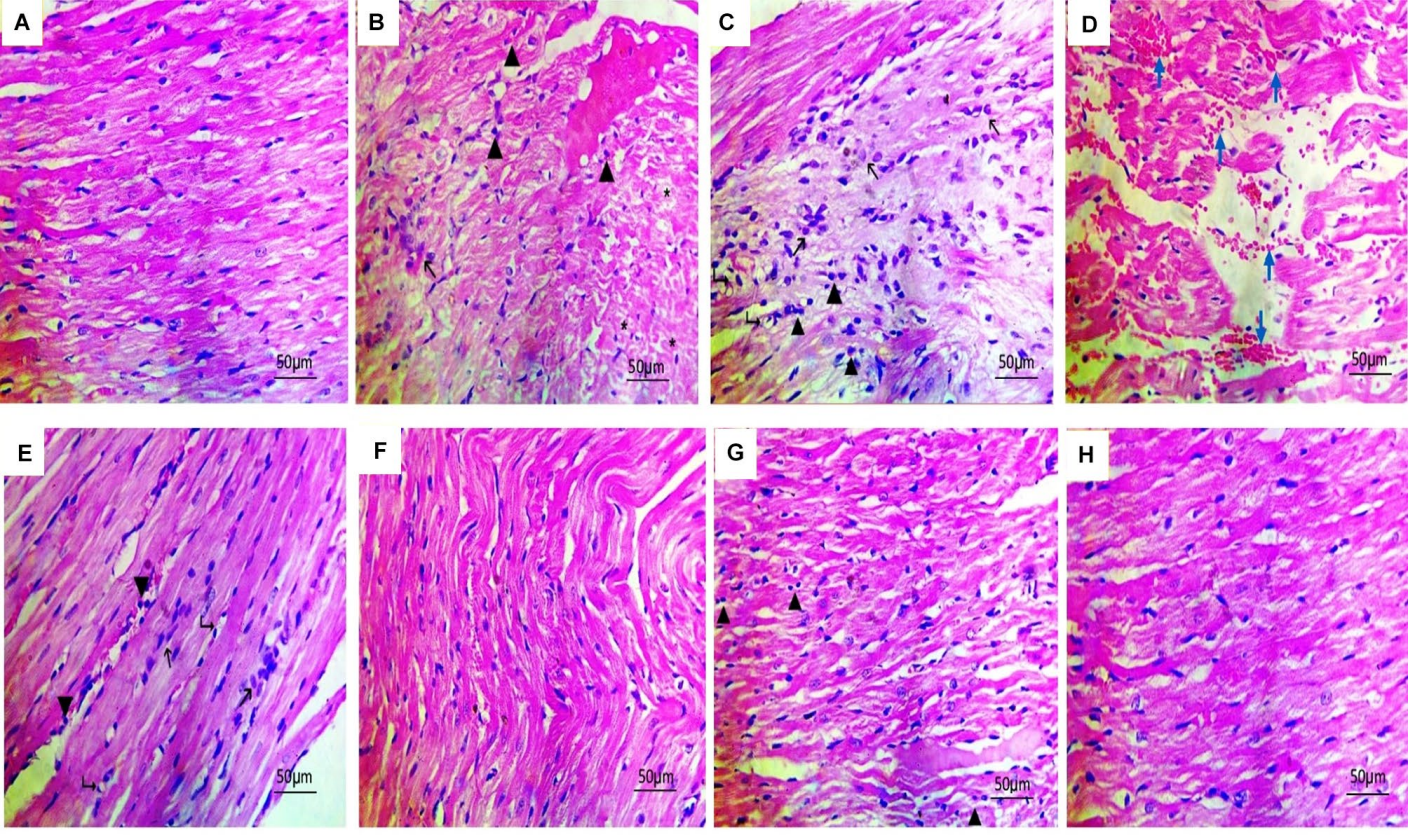
Effect of Br-MSC, metformin, and their combination on cardiac histopathological sections. (**A**) The myocardium section of the control rats showed normal structure and thickness of the cardiac muscle (**B–E**) Heart sections of cisplatin-treated rats showed wide areas of myocytes degeneration (asterisks) replaced by fibroblasts (black arrows) and inflammatory cells (arrowheads) with vacuolated blood vessels (Rt. sided arrowheads) and separation of myocytes by extravasated RBCs (blue arrows) with loss of muscular striations. (**F**) Heart section of Br-MSC treated animals showing improvements of myocyte necrotic changes with nearly normal cardiac muscles (thinning with some fibers disarray) (**G**) Heart section of the metformin-treated group showing improvement of the myocyte’s necrotic changes (thinning with some fibers disarray) with infiltration some inflammatory cells (upward arrowheads) (**H**) Heart section of Br-MSC + metformin treated rats revealing marked improvements of the myocytes with normal structure and thickness. All sections were stained with H&E (× 400) (scale bar = 50 μm).

The heart sections of cisplatin-treated rats showed areas of myocyte degeneration and myofibrillar loss that were replaced by fibroblasts with loss of muscular striations. Furthermore, there was widespread inflammatory cell infiltration between the muscle fibers. On the other hand, normal structure and thickness of cardiac muscle are seen in the cardiac sections of the control animals. Rats treated with Br-MSCs or metformin revealed an improvement in the integrity of the cardiac cells as there is an absence of myocyte degeneration with a marked reduction in inflammatory cell infiltration. The representative cardiac sections of rats treated with the combination of Br-MSC and metformin showed marked improvements with nearly normal cardiac tissue (Fig. 4, Table 4).

**Table 4:** Semi-quantitative scoring showing the severity of changes in cardiac muscle and the effect of Br-MSCs, metformin, and their combination on cardiac histopathological sections.

Hisopathological changes	Control	Cisplatin treated	Br- MSCs + cisplatin	Metformin + cisplatin	Br- MSCs + metformin + cisplatin
Disruption of cardiac muscles architecture	**–**	** + + + **	** + **	** + **	** ± **
Loss of muscular striations	**–**	** + + + **	** + + **	** + **	**–**
Myocyte degeneration	**–**	** + + + **	** + **	** + **	** ± **
Fibrosis	**–**	** + + **	** + **	** + **	** ± **
Inflammatory cellular infiltrate	**–**	** + + + **	** ± **	** + + **	**–**

Scores were expressed as follows, (–) normal; ( ±) borderline; ( +) mild; (+ +) moderate; (+ + +) severe.

## Discussion

Even though cisplatin causes high cancer cure rates, the cardiotoxic hazards are still an aggravating restriction to its clinical use^38,39^. Distinct agents were investigated to protect the heart from cisplatin’s deleterious effects, revealing various outcomes^2,40^**.** Due to the wide spectrum of cisplatin cardiac side effects varying from mild to severe ones, the demand to look for effective treatment tools to overcome this concern is increasing^41^. The current study was conducted to investigate the effects of Br-MSC, metformin, and their co-administration with insights into mechanism-based protection against the cardiotoxic effects of cisplatin. The study demonstrated that treatment by Br-MSCs and metformin lowered the high serum cardiac enzyme indices, decreased the oxidative stress malondialdehyde (MDA) levels, restored the antioxidant superoxide dismutase (SOD) capacity, inhibited cardiac pro-inflammatory cytokines interleukin-1ß (IL-1ß) and tumor necrosis factor-α (TNF-α) production, lowered cardiac pro-apoptotic Bax gene expression and increased the anti-apoptotic Bcl-2 gene expression, as well as AMPK activation, with the improvement of myocytes histological derangements worsened by cisplatin injection.

Br-MSCs have developed a massive turnover in treating many disorders because of being more active with a higher differentiability index than other stem cell types^14^. In the present work, the intraperitoneal infusion of Br-MSCs enables them to migrate to the injured cardiac tissue escaping the pulmonary trapping that characterizes the intravenous route^42^. Similar studies have endorsed the homing ability of stem cells following systemic intraperitoneal administration^14,43^. This could be attributed to the reaction and adhesion between the chemokines, cytokines, and growth factors released from injured cardiac tissues with their surface ligands on Br-MSCs with subsequent transmigration across the endothelium^44,45^.

In the present study, treatment with cisplatin significantly decreased heart weight which is accompanied by a parallel change in the ratio between heart weight to body weight. These results may be driven by cardiac tissue necrosis as seen in histopathological pictures which agree with Shaker and colleagues^46^ work. However, Bayrak et al.^47^ while studying the possible effects of acetyl-l-carnitine against cisplatin-induced cardiotoxicity, found no significant difference in heart measurements and weights among the studied groups. On the other hand, these findings were prevented after treatment with Br-MSCs, suggesting that Br-MSCs can halt the cardiac gross anatomical changes driven by cisplatin cardiotoxic effects.

In agreement with Saleh et al.^4^ and Hu et al.^5^ studies, our study showed that cisplatin injection was accompanied by worsening in the cardiac functions as documented by the alerted ECG pattern evidenced by increased heart rate, elongation of QTc interval duration with increased ST segment height and T wave amplitude as compared to normal ECG. These findings were in line with the elevated cardiac function parameters as serum troponin T and CK-MB activities thus denoting marked cardiac injury.

Even though the mechanisms of cisplatin cardiac toxicity are not clear, research has confirmed the role of oxidative stress and reactive oxygen species (ROS) generation^48^. The overproduced oxidative radicals after cisplatin administration react with cell membrane lipids, proteins, and nucleic acids evoking severe tissue damage^49^. In the present work, the high levels of MDA along with the decreased enzymatic activities of SOD are proof of the oxidative stress caused by cisplatin injection. These findings are in accordance with the work of Qian et al.^39^ and Bayrak et al.^47^. The decrease in SOD levels could be attributed not only to ROS generation but also to the renal loss of copper and zinc, which are essential for SOD enzyme activity^6^. Nevertheless, Br-MSCs significantly ameliorated the cardiac functional and structural damage proven by a significant reduction in the myocardial enzymes, oxidative stress, inflammatory cytokines, and pro-apoptotic markers. Liu et al.^50^ attributed the regeneration of the damaged myocardium after MSCs therapy to their ability in mitigating ROS-induced apoptosis.

Studies have demonstrated the role of inflammation as another accepted mechanism of cisplatin cardiac toxicity^51^ and the close interrelation between oxidative stress and inflammation could worsen this cardiotoxicity^52^. Consistent with the work of Xing et al.^51^, the present study demonstrated that cisplatin intoxication significantly increased the expression of the cardiac inflammatory mediators TNF-α and IL-1ß, this was because they were harmonious with the deteriorated antioxidant defense and augmented oxidative stress. Indeed, in response to inflammation, the production of various pro-inflammatory and pro-oxidative enzymes results after the activation of neutrophils, thus inducing severe tissue damage^53^. Furthermore, Zhang et al.^54^ suggested that the exaggerated cellular stress by various oxidants is the main leading cause of the high levels of pro-inflammatory cytokines such as TNF-α, IL-1, and IL-6. On the other hand, our results denoted that injection of Br-MSCs mitigated the levels of pro-inflammatory cytokines. Interestingly, Ries and co-workers^55^, stated that the pro-inflammatory cytokines, IL-1ß and TNF-α, released from the injured tissues up-regulated the matrix metalloproteinases in MSCs leading to their chemotactic migration through the extracellular matrix. Meanwhile, the anti-inflammatory potential of the MSCs could also explain the remolding of the heart tissue not only via regulating the natural killer cells, B and T cells, macrophages, and neutrophils but also via secreting various immune regulators, such as interleukins (IL-4, IL-6, IL-10), transforming growth factor-beta (TGF-ß), prostaglandin-E2 (PGE2)^56^. Furthermore, it was found that MSCs can secrete various paracrine factors that have different properties including proliferative, anti-apoptotic, angiogenic, and anti-fibrotic properties^57,58^. However, co-administration of metformin with Br-MSCs indicates an almost complete recovery from using Br-MSCs alone which is evidenced by ECG pattern improvement, and normalization of cardiac enzymes that is further supported by the histopathological picture improvement. This agreed with the work of A Soliman et al.^44^ on the doxorubicin cardiotoxicity rat model who found that sodium valproate had improved bone marrow mesenchymal stem cells (BM-MSCs) survival compared to BM-MSCs injection alone or sodium valproate treatment alone.

Accumulating evidence suggests that myocardial apoptosis is an inevitable sequence after cisplatin injection. Qi, et al.^59^ have reported cardiomyocytes apoptosis in cisplatin-treated mice by the obvious increase in the activity of caspase-3 and the expression of the pro-apoptotic proteins BAX and BAK, in addition to the diminished expression of the antiapoptotic markers, Bcl-2, Bcl-XL. In accordance with Qi and colleagues^59^ study, cisplatin has lowered cardiac pro-apoptotic Bax gene expression and increased the anti-apoptotic Bcl-2 gene expression in the current work. Nevertheless, Br-MSCs significantly restored Bax/Bcl-2 Ratio. The previous studies imputed the cardiac apoptosis to the generation of ROS as a byproduct of cisplatin metabolism, as the triggered lipid peroxidation cause cardiomyocyte membrane damage with activation of DNA damage markers and eventually myocyte apoptosis or necrosis^60,61^. All the evidence shows the potential protective role of Br-MSCs in the reverse of cisplatin-induced cardiac apoptosis by inhibiting ROS-mediated DNA damage.

Earlier investigations have demonstrated the role of mitogen-activated protein kinases (MAPKs) as a pro-survival pathway in cardiomyocyte proliferation^26,62^**.** In the present study, cisplatin significantly downregulates AMP-activated protein kinase (AMPK) gene expression in the cardiac tissue, which is consistent with Zhang et al.^6^ who studied the role of apelin-13 on cisplatin-induced cardiotoxicity and confirmed the protective role of PI3K/AKT, ERK, and AMPK signaling pathways in amelioration of the cardiac injury induced by cisplatin. Furthermore, Cisplatin through PI3K/Akt/GSK-3β signaling pathway evoked mice cardiotoxicity in Xing et al.^51^ studies. On the other hand, treatments with Br-MSCs and metformin have restored these results.

Metformin, an oral biguanide agent, has been used for many decades in the treatment of diabetes; however, its cardioprotective role has been confirmed in both diabetic and non-diabetic people^63,64^. In the present study, metformin was found to ameliorate the cardiac enzyme indices and the elevated oxidative stress and inflammatory markers which agrees with Loi et al.^64^. Many mechanisms have been involved in the cardioprotective actions of metformin including inhibition of the production of ROS, attenuation of mitochondrial damage with the maintenance of energy production, and activation of AMPK^26^. Various studies supported the role of AMPK in metformin cardioprotective mechanisms either in cultured cells or in animals^24,26,62^. The antioxidant, anti-inflammatory, and anti-apoptotic properties suggest that metformin could offer protection against cisplatin cardiotoxicity, and not surprisingly that its co-administration with Br-MSCs in the current work has superior results than using each treatment alone.

## Conclusion

In conclusion, our research revealed the promising protective potential of Br-MSCs in mitigating cardiac insults evoked by cisplatin administration evidenced by improved cardiac indices, amelioration of oxidative stress, and reduction of pro-inflammatory cytokines and apoptotic markers with histopathological derangements improvement. The possible underlying protective mechanism may be partly attributed to the inhibition of oxidative stress, inflammation, and apoptosis via the AMPK pathway.

The limitation of our study was that we did not investigate which molecule activates AMPK and whether there are any potential targets for the protective effect of Br-MSCs. Therefore, more research studies are needed to focus on other molecules upstream and downstream of the AMPK signaling pathway.

## Supplementary Information

Supplementary Information 1.

Supplementary Table S1.

## Data Availability

The generated and analyzed datasets of the current study are available from the corresponding author upon reasonable request. The gene expression data analyzed during the current study are available as supplementary data.
