# Supplementary material for: Breast milk mesenchymal stem cells abate cisplatin-induced cardiotoxicity in adult male albino rats via modulating the AMPK pathway
Source: Sci Rep. 2022 Oct 20;12:17554. doi: 10.1038/s41598-022-22095-2 (PMC9585145; doi:10.1038/s41598-022-22095-2)
Supplement: Supplementary file 2 — Supplementary Table S1. [file 41598_2022_22095_MOESM2_ESM.docx]

# Breast milk mesenchymal stem cells abate cisplatin induced cardiotoxicity in adult male albino rats via modulating the AMPK pathway

# Mahitab M. Nageeb^1^, Sara F Saadawy^2^, Seba Hassan Attia^1^*

# ^1^ Clinical Pharmacology Department, Faculty of Medicine, Zagazig University, Zagazig Egypt

# ^2^ Medical Biochemistry Department, Faculty of Medicine, Zagazig University, Zagazig Egypt

***Corresponding author:** Seba Hassan Attia

***Affiliation****:* Clinical Pharmacology, Faculty of Medicine, Zagazig University, Egypt, ***e-mail:*** [SHAttia@medicine.zu.edu.eg](mailto:SHAttia@medicine.zu.edu.eg).

**Supplementary table**

**(S1): Primer sequences used for quantitative real-time PCR assays**

| **Gene** | **Forward primer (5′–3′)** | **Reverse primer (5′–3′)** | **Accession No** | **Product size** |
| --- | --- | --- | --- | --- |
| GAPDH | GGCACAGTCAAGGCTGAGAATG | ATGGTGGTGAAGACGCCAGTA | NM_017008.4 | 143 |
| Bax | CGAATTGGCGATGAACTGGA | CAAACATGTCAGCTGCCACAC | NM_017059.2 | 109 |
| Bcl-2 | GACTGAGTACCTGAACCGGCATC | CTGAGCAGCGTCTTCAGAGACA | NM_016993.1 | 135 |
| AMP-activated protein kinase (AMPK) | ACACCTCAGCGCTCCTGTTC | CTGTGCTGGAATCGACACT | NM_023991 | 67 |

**GAPDH**: Glyceraldehyde-3-phosphate dehydrogenase
